# Communication of bed allocation decisions in a critical care unit and accountability for reasonableness

**DOI:** 10.1186/1472-6963-5-67

**Published:** 2005-10-31

**Authors:** Andrew B Cooper, Amit S Joglekar, Jennifer Gibson, Alissa H Swota, Douglas K Martin

**Affiliations:** 1 Department of Critical Care Medicine, Sunnybrook and Women's College Health Science Centre and University of Toronto, Toronto, Canada; 2 Department of Anaesthesia, University of Toronto, Toronto, Canada; 3 Joint Centre for Bioethics, University of Toronto, Toronto, Canada; 4 Department of Philosophy, University of North Florida and Blue Cross Blue Shield of Florida Center for Ethics, Public Policy, and the Professions, Jacksonville, USA; 5 Department of Health Policy, University of Toronto, Toronto, Canada

## Abstract

**Background:**

Communication may affect perceptions of fair process for intensive care unit bed allocation decisions through its impact on the publicity condition of accountability for reasonableness.

**Methods:**

We performed a qualitative case study to describe participant perceptions of the communication of bed allocation decisions in an 18-bed university affiliated, medical-surgical critical care unit at Sunnybrook and Women's College Health Sciences Centre. Interviewed participants were 3 critical care physicians, 4 clinical fellows in critical care, 4 resource nurses, 4 "end-users" (physicians who commonly referred patients to the unit), and 3 members of the administrative staff. Median bed occupancy during the study period (Jan-April 2003) was 18/18; daily admissions and discharges (median) were 3. We evaluated our description using the ethical framework "accountability for reasonableness" (A4R) to identify opportunities for improvement.

**Results:**

The critical care physician, resource nurse, critical care fellow and end-users (trauma team leader, surgeons, neurosurgeons, anesthesiologists) functioned independently in unofficial "parallel tracks" of bed allocation decision-making; this conflicted with the official designation of the critical care physician as the sole authority. Communication between key decision-makers was indirect and could exclude those affected by the decisions; notably, family members. Participants perceived a lack of publicity for bed allocation rationales.

**Conclusion:**

The publicity condition should be improved for critical care bed allocation decisions. Decision-making in the "parallel tracks" we describe might be unavoidable within usual constraints of time, urgency and demand. Formal guidelines for direct communication between key participants in such circumstances would help to improve the fairness of these decisions.

## Background

While there are consensus documents describing how decisions about ICU admission should be made and epidemiological data about the characteristics and outcomes of patients referred but refused admission [1,2] there is little information about this complex decision making process in the context of resource scarcity and unrelenting demand. Fairness in limit setting for admission to the intensive care unit can be evaluated using "Accountability for Reasonableness" (A4R). In the A4R framework, a fair process supplements moral deliberation based on substantive criteria and moral principles to guide resource allocation [3]. Priority setting in health care institutions is considered fair and legitimate if it satisfies four conditions (Table 1). When A4R was used to evaluate fairness of priority setting in two previous studies of intensive care units, deficiencies in the publicity and appeals conditions were observed [4]. The role of communication in the process of limit setting and its relationship with the publicity condition was highlighted in both studies, however a commentator noted "an incomplete picture of the chain of evidence" leading to the conclusion that there should be greater transparency to improve fairness [5].

**Table 1:** The four conditions of accountability for reasonableness

Publicity	The decisions and reasons behind priority-setting decisions must be publicly available.
Relevance	These rationales must rest on evidence, reasons, and principles that fair-minded people can agree are relevant to deciding how to meet the diverse needs of patients in the context of limited resources.
Appeals	There is a process for revision and dispute resolution regarding priority-setting decisions.
Enforcement	There is a method of regulation in place to ensure that the first three criteria are met.

We sought to describe participants' perceptions of communication during the priority-setting process in the Critical Care Unit (CrCu) of Sunnybrook and Women's to gain an understanding of how the fairness of our bed allocation process might be improved.

## Methods

### Design

Qualitative case study research was used to describe priority setting at the microallocation level. This is the appropriate method for investigating a complex social phenomenon in its real life context [6].

### Setting

Our study was conducted at Sunnybrook and Women's College Health Science Centre in its busy critical care unit (CrCu). Beds in CrCu are available to Sunnybrook hospital programs; Surgical Oncology, Trauma, and Community. From the unit census, 1100 medical, trauma and surgical patients were admitted in 2001–2002. The median number of occupied beds at daily census in the critical care unit during the interview period (January – April 2003) was 18/18 with a median of 3 admissions and discharges per day.

### Sample

Theoretical sampling was used in choosing "key" participants and documents. We conducted 18 semi-structured interviews with people directly involved in microallocation in our critical care unit and we examined the content of three relevant hospital policies. We examined documents if interviewees made use of them to support decision-making. We included individuals who were recommended to us in previous interviews. For example, if an interviewee told us that resource nurses had a role in bed allocation, we interviewed resource nurses. The interviewees consisted of 3 critical care physicians, 4 clinical fellows in critical care, 4 resource nurses, 4 end-user physicians who commonly referred patients to the intensive care unit and 3 members of the administrative staff. We continued interviewing until we reached saturation; a point at which no new concepts or important actors in the process were identified in subsequent interviews.

Bed allocation in the critical care unit is specified by a policy *"Critical Care Directorate [CrCu] Resource Prioritisation" *which addresses the ICU needs of the following institutional programs; Trauma, Surgical Oncology and Community. This policy specifies priorities for intensive care unit admission as follows: 1.) Intramural patients; any program affiliation, including war veterans from an adjoining chronic care facility, 2.) Extramural trauma referrals [as long as one intensive care unit bed remains available] and surgical oncology patients (up to a maximum of 2 occupied intensive care unit beds per day), 3.)Extramural referrals including neurosurgical patients and other elective surgical patients requiring postoperative care in the intensive care unit. The critical care physician in charge is empowered by policy "*VI-A-10 Admission of Acute Care Patients: Section 6 *– *Admission to a Critical Care Unit. Priority of Admission to a Critical Care Unit*" as the sole participant making bed allocation decisions. Disputes or concerns about intensive care unit bed allocation decisions, such as their potential to compromise patient safety due to inadequate resources are arbitrated by the Medical Director on Call whose authority to over-rule decisions is established by policy "*Core Patient Care Policy: Patient Flow"*.

### Data collection

Interviews consisted of open-ended questions related to priority setting and fairness of the decision-making process (Table 2). The interviewer asked further specific questions to clarify answers and focus the subject on the question that was asked. The interview was audio taped and then transcribed verbatim. The transcripts were read and analysed by two individuals (AJ, AC). Three policy documents that pertained to admissions and discharges from the ICU were identified and analysed.

**Table 2:** Interview guide

How do you decide who gets an ICU bed?
Who is involved in making these decisions?
To whom are these decisions communicated?
Describe an example when this was a very difficult decision?
What happens if someone wants to appeal or challenge a decision?
How does making these decisions, in the way you have described, make you feel?
Do you think that the way decisions are made is fair?
What resources are available to help your decision-making?
Are priority-setting decisions consistent with the guidelines?
Who else should I talk to about this?

### Data analysis

Modified thematic analysis of the interviews and documents involved reading through the transcribed data and identifying concepts that related to how priority-setting decisions were made. Conceptually associated text was organised in Microsoft Excel© spreadsheets labelled with descriptive categories. We grouped data with similar concepts into overarching themes related to allocation decisions, and then sorted it according to the identified key participants We then extracted data describing the process of communication involving these participants, focussing on content and the communication methods used. Finally we used writing to formalise our interpretation of concepts emerging from the data, and to make our interpretations explicit.

### Ethics approval

The study was reviewed and approval was granted by the research ethics committees of Sunnybrook and Women's and the University of Toronto. No patients were interviewed.

## Results

We will describe the perspectives of those involved in the bed allocation decisions, showing their perceptions of the lines of communication, its usual methods and content.

### Critical care physicians

Critical Care physicians communicated their decisions to the Medical Director On call (an administrator empowered by policy to adjudicate controversial decisions), Trauma Team Leader, Resource Nurse, Operating Room, Surgical House staff, and Surgeons. Most critical care physicians in our sample did not identify a need to communicate their decisions to families of patients cared for by end-users.

*Depending upon how full we are in the ICU I may need to communicate directly with the overhanging users of the ICU *... *to say, sort of, "the ICU is getting full," or, "is full and we are really be hard [sic] to accept any more traumas," so they know not to accept if we can't care for patients. As well, the operating room, other than the surgeons to say, you know, "this is how we're doing, we are not going to be able to accommodate the patient that was scheduled to come here*.

When communicating with end-users, Critical Care Physicians discussed reasons for being unable to agree to a request for an ICU bed. They included program-based prioritization, (e.g. surgical oncology would have precedence over surgical services not identified as high priority), lack of capacity, and anticipated or approaching lack of capacity. Some physicians communicated reasons for denying a bed directly to end-users, while others noted that they might communicate indirectly via house staff or by leaving a message with the receptionist at the operating room desk for the surgeon.

if a surgeon has a patient booked, the OR desk and the operating room that the surgeon would be working in that day would be advised, it might not necessarily be the surgeon directly, the OR desk may say put room 1 on hold because we are not sure about the bed; but the message gets to the surgeon who has booked the case that way

**Figure 1:**
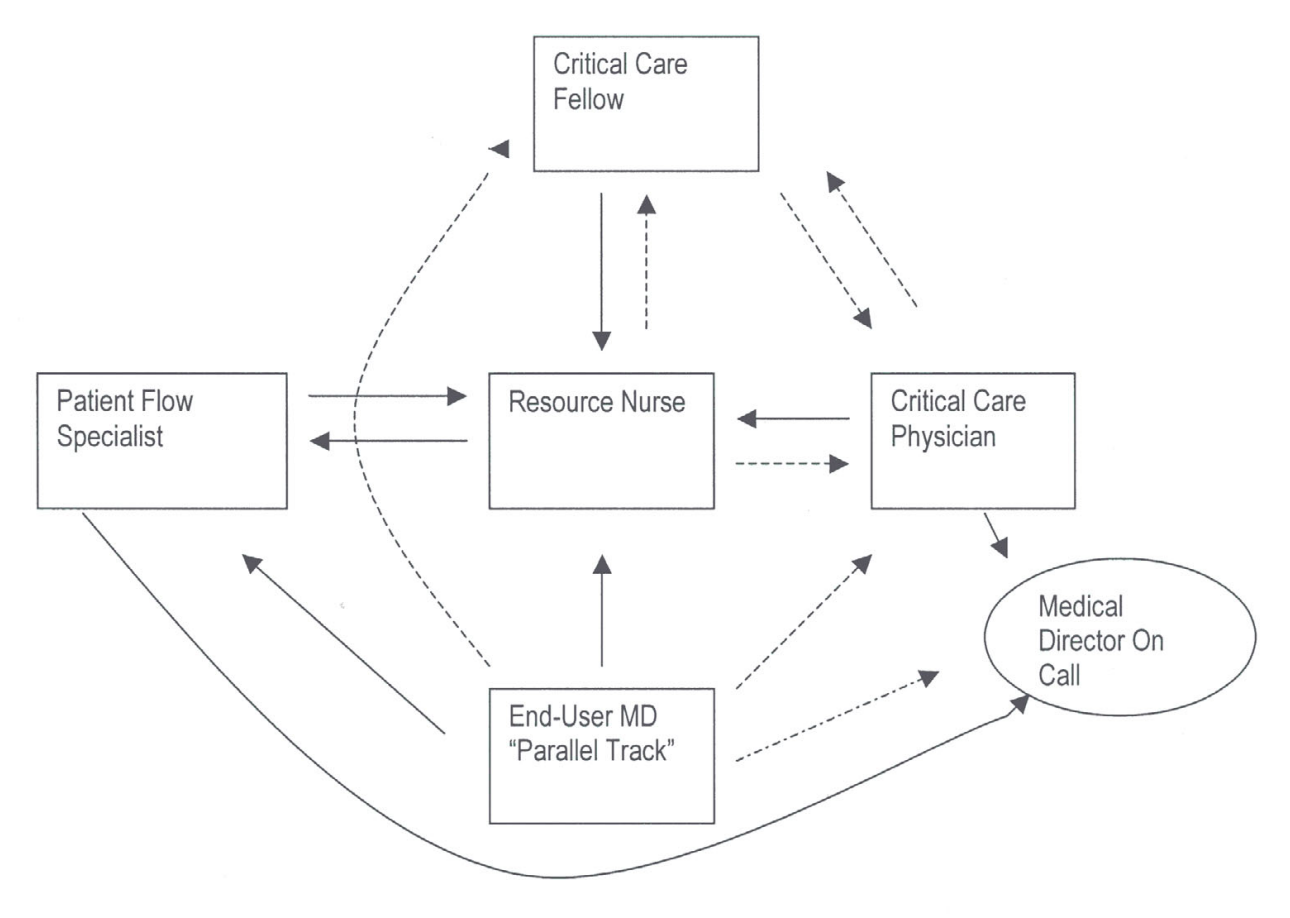
Communication of ICU Bed Allocation Decisions. Communication involved in intensive care unit bed allocation decision-making is indirect. "End user physicians" speaking directly to the resource nurse, but not the critical care fellow or attending physician, constituted a "parallel track " of bed allocation decision-making. **Arrows **-direction of communication between participants **Solid Lines**- consistent communication of need for intensive care resource. **Broken Lines**- inconsistent communication of need for intensive care resource.

Critical care physicians required direct communication of requests for beds. In harmony with the policy *VI-A-10 Admission of Acute Care Patients: Section 6 – Admission to a Critical Care Unit. Priority of Admission to a Critical Care Unit *they viewed this communication as a necessary condition for any end-user to access an ICU bed. Although required by policy, direct communication was often omitted in practice. The indirectness of communication between the critical care physician and end-users resulted in unofficial "parallel tracks" of allocation decision-making (see below and Fig. 1). The lack of direct communication between the critical care physician and end-users could result in each being unaware of the other's bed allocation decisions and rationales.

### "End user" physicians

We use the term "end-user physician" to designate participants from any hospital program. Our end-user participants included physicians from medical and surgical services who sought access to the ICU resource on behalf of their patients. End-user physicians discussed ICU bed allocation decisions with critical care physicians, clinical fellows and house staff, the ICU resource nurse, the surgical oncology triage coordinator, bed care coordinators, extramural referring services, and patients or families. End-Users perceived the critical care fellow or resource nurse as good sources of information, but the availability of a bed could be better ascertained from a discussion with the resource nurse.

*I've found some fellows when I talk to them, they've been happy to make the decision on their own and that may reflect a discussion that they've already had with the attending. And other times the fellow will say, "well this is something I need to clear with the attending. I think its ok but let me check and I'll get back to you." So at times they act as a go between other times they make the decision and I don't know if that decision is one that's already made beforehand or they're at a senior enough level or comfortable enough to, you know, so it's variable*.

At Sunnybrook and Women's, clinical fellows communicate with critical care physicians about proposed admissions to the units, but they are not permitted to allocate beds independently. Reflecting this, some end-users bypassed the clinical fellows completely and based their possibilities for access to available beds on discussion with the ICU resource nurse. One respondent perceived that this information was enough to allow a decision to be made to accept an extramural referral.

*On the other hand if there are beds, you're given the situation when there's 6 beds, and then I don't really have to talk to anybody I just have to tell the resource nurse and they're happy usually to "ok" the decision*.

End users perceived that communication with the critical care physician would clarify rationales when patients were refused admission.

*human nature being what it is, if the answer to you is "no you don't get a bed" than usually you're going to ask why and usually they try to give you a bit of a rationale as to why you didn't get the bed and "Sid Viscious " got the bed*.

However, they perceived that communication with the critical care physician was less than ideal. Critical care physicians could be vague about the status of bed availability.

there's this frustration of, you don't get a straight answer, "well maybe we have a bed," "well when will you know?" "Well, give us an hour."

There was variability in the response of individual critical care physicians to families about the rationales for refusal of admission. Some end-users felt this was not inappropriate.

well hopefully, hopefully, they would communicate it to everyone involved. I think in practice its communicated with either the resident or the staff and may or may not discuss it with the family. the ICU doc has no previous relationship with the family member so it may be more appropriate for the primary care doc to go back to the family

When elective surgery requiring postoperative intensive care was affected by bed shortage, end-users identified a mediator; the Surgical Oncology Triage Co-ordinator who would intervene to decide which of two surgeries requesting the same bed would proceed.

*on a given day, the ICU may say to us, "you know what, you're allowed 2 beds but we don't have 2 beds, we have one bed or we have zero beds," so then you have to fight with yourselves and what they do is say that the two surgeons who have beds will fight with themselves or there is a coordinator who has to decide and generally the ICU tries to keep itself out of that discussion*.

### Resource nurse

The resource nurse and critical care physician approve proposed admissions after considering their impact on physical and human resources in the intensive care unit.

It's more the resource nurse and the doctors along with the bed flow person, really, because I get them involved, you know, you've gotta get them involved, because you've gotta know how your bed situation goes. So right of the bat, in the morning I call them, "I've got three people to go out, how's the beds looking?"

The central role of the resource nurse in ICU bed allocation is seen in the diversity of stakeholders with whom she must communicate; the critical care physician, critical care fellows, trauma team leader, operating room, referring services and patient flow specialist. Combined with end -user perceptions that the resource nurse could make bed allocation decisions, this resulted in the resource nurse assuming an unofficial (contrary to policy) role as the primary decision maker.

*the patient was gone to the OR and I took their bed and just hoped that they'd be in the OR for the same time as the other patient was going to organ procurement and this patient was coming back from the OR. That was, I didn't really go through anybody to make that decision*.

*we hear from an anaesthetist in the OR, "we're having trouble, it doesn't look like this patient is going to be extubatable, is there a bed?" If there isn't a bed we usually say "can they go to recovery room, and then we'll assess them later?" Or sometimes they say adamantly "no, they have to come." Then usually I just let the fellow or staff man know*.

Communication of bed allocation decisions was verbal, informal and typically occurred over the telephone. Resource nurses played a key role in identifying breaks in the flow of information that might exclude participants, such as the critical care physician from involvement in the decision making process.

*By phone and it starts with the resident or the fellow and it doesn't usually go straight to the staff. Usually. And sometimes they call me; they'll call the resource nurse looking to see if there is a bed or if there'll be bed*.

For priority hospital programs with special entitlement to ICU resources, communication of bed requests might be made with very little direct communication between the ICU participants and the progam's end-user physicians. This was especially true for the trauma program. Communication between the trauma program and the resource nurse mirrored the indirectness already described in communication between the critical care physician and end-users.

*traumas usually make their way through the resource nurse and the resource nurse usually tells the fellow that there's a trauma on its route and that's how that one is communicated*.

*if it's a trauma patient it could be the ward clerk down in emerg who'll just call and tell us there's a trauma coming, like eta or whatever and if they're tubed or not. Usually when they tell us that they're tubed we just automatically say, "well ok they're going to need to come to the ICU"*.

### Clinical fellows

Clinical fellows communicated ICU bed allocation decisions with team-members, including the critical care physician, resource nurse, on-call resident and bedside nurses. They also discussed decisions with stakeholders external to the ICU; referring services, patients and their families.

*I guess to start with, the people taking care of the patient wherever they are, so the nurse and the resident physician. I don't always talk to the staff directly. I don't often deliver it to the attending on the floor. The patient, the patient's family if they're available, if not maybe a little later on then the patient's family. The resource nurse in the ICU, the resident that I'm on call with, and eventually after the patient*... *the staff that's on call; once they're all monitored and everything*.

Clinical fellows reviewed requests for ICU beds and their appropriateness (in relation to admission criteria) with referring services. Clinical fellows were the only participants who described discussing reasons for non-admission with patients or families when patients were deemed inappropriate for intensive care. However, not all clinical fellows we interviewed described doing this, and some respondents were uncomfortable with the practice.

*when the ICU says, "there is no role for ICU care here", and the patient's family thinks that there is and in that situation first I would try talking to both the patient and the family*.

*Saying to someone that well "you're low priority as compared to another patient who's sick elsewhere," seems kind of wrong*.

Discussion of referrals with critical care physicians was usually done directly. Clinical fellows described contacting referring service superiors if there was disagreement about their decisions not to admit a patient to the ICU. However, they commented on feeling left out of decisions made by other participants to allocate beds; fellows found that they needed to take the initiative to remain informed.

*If my staff decides to bring somebody in the unit, they always tell the charge nurse and they usually tell me, they don't always tell the house staff. If the charge nurse decides to bring somebody into the unit they usually talk to the staff but they don't often inform the house staff or the fellow. So that's why, like when I first come in, in the morning, I go to the charge nurse and find out how many flow throughs we're having from the OR, cause otherwise you don't know how many beds you need to have empty and then I go to them, essentially, every 2 hours and say, "are there any other patients you've heard of, are there any traumas in the wings, are there any criticall patients coming, is anything going wrong in the OR, cause they're the person who will have that information but they don't necessarily tell all the house staff*.

Clinical fellows observed that communication of bed allocation decisions to end-user physicians and families of patients affected by them were without formal guidance. Clinical fellows, like end-users observed that the reasons for bed allocations were not always accessible.

it happens when outside services really want somebody to come into the unit, I mean we've initially said no and their staff is more that welcome to call my staff and discuss it and come to some amicable agreement but usually at that stage the fellows and the house staff are usually out of the decision making process

### Administrators: patient flow specialist

Patient flow specialists monitor resource availability (critical care and other beds) within the institution and communicate this to the critical care physician, end users and resource nurse.

sometimes it is definitely a collaborative effort between all the different physicians and sometimes nursing input, often patient flow will be called to see, where the pressure points are "can we do this, can we take another patient, can you get people out so that we can make this happen?"

When disagreements arise about interpretation of prioritization rules outlined by policy, the patient flow specialist is obligated by policy to communicate with the medical director on call. The role of the medical director was to direct physicians to decline transfers and to cancel or reprioritize surgery. This involvement was sought when the patient flow specialist was unable to convince those requesting a bed that the proposed ICU admission would be unsafe.

if there's a problem with someone doing something like... pulling someone into critical care or doing something extraneous to get people out of there which is going to impact the hospital or hurt someone. We can say "no" and we can pull a physician in to talk to them immediately.

Patient flow specialists' perceptions of the communication in bed allocation decision making included an appreciation of its complexity and urgency. Communication of these important decisions was ad hoc and lacked formal procedural guidance.

*there isn't any sort of formal mechanism that we must make sure on the tick sheet that this person contacts this person, this person, it just happens form a what makes sense point of view for the situation*.

## Discussion

Our case study is the first to concentrate on the communication of critical care unit bed allocation decisions. We found a complex interaction between multiple participants in which information impacting on decisions to allocate beds was exchanged (Table 3, Figure 1). However, the methods of communication were often indirect, sometimes with complete breaks in the flow of information between key participants. Communication breaks often impacted those most directly affected by a decision not to allocate a bed, such as the end-user surgical oncologist or the families of patients requiring intensive care treatment. These results are important because they suggest ways to improve the publicity of limit setting decisions in critical care units.

**Table 3:** Communication of ICU Bed Allocation Decisions

Multiple Decision Makers	Critical Care Physician Intensive Care Unit " Resource Nurse" End-User Physicians Critical Care Fellows Patient Flow Co-ordinator
Independent Functioning	''Parallel tracks'' of decision-making
Indirect Communication	Telephone Intermediaries [housestaff, receptionists]
No Guidelines	Affected parties left "out of the loop"

In a study of priority setting in a large university affiliated teaching hospital, Mielke et al found multiple participants in the decision making process [4]. Further to the key participants we identified, these investigators found members of the hospital medical advisory committee, legal counsel and bioethicists as participants. This finding may reflect differences in scope between our investigation and theirs; we did not extend our investigation to committee meetings. Arguments have previously been made for nurses to be advocates for care preferences and operational safety within the process of decision-making [7,8]. We found that key decision makers talked to the resource nurse during their evaluation of the availability of intensive care unit beds. The prominent involvement of some nursing personnel in critical care unit bed allocation is a unique finding of the present study. This implicit acknowledgement of the importance of the resource nurse in our intensive care unit contrasts with survey findings that nurses perceive their input is not valued [9]. The perception that a bed was available after discussion with the resource nurse was sufficient for some participants to make a commitment to admit their patients to the unit, even without further attempts to communicate with the official gatekeeper, the critical care physician.

Adding to the complexity of the process we studied were "parallel tracks" of decision-making. End-user physicians (neurosurgeons, operating room surgeons and anesthesiologists), the critical care unit resource nurse, and the most entitled service (trauma program) all identified circumstances in which they might make independent bed allocation decisions. This may explain why some end-user physicians experienced frustration in their attempts to access critical care unit resources. While official accountability for bed allocation decisions rested with the critical care physician, "parallel track" decision makers could allocate beds without this participant's knowledge. Previous investigators have demonstrated that parallel track decision makers may view the critical care unit physician as responsible for making decisions about the appropriateness of discharges, but may not consult with them about decisions to admit patients [10]. This exclusion from the decision making process may cause the official gatekeeper to be unable to explain why beds are not available in the critical care unit at a given time. When ICU beds are readily available, decision makers find it difficult to deny patient access to a bed- the " non triage mode". When there is a high census or full occupancy, decision makers must prioritize admissions to the ICU- "triage mode". Involvement of the ICU physician in triage mode involves reviewing and prioritizing proposed admissions and decision making about discharges of patients who can safely be discharged or transferred. Review of patients who are not responding or benefiting from continuing intensive care also occurs [11]. "Parallel-track" decision-making resulting in ICU bed allocation occurred in both modes in our case study. When ICU beds are readily available, parallel – track decision makers such as the resource nurse, clinical fellow or end user physicians might reasonably make bed allocation decisions based on ICU admission criteria, improving the responsiveness and flexibility of the resource. However, the importance of direct communication between parallel – track decision makers and the critical care physician – gatekeeper is not diminished because admissions in the "non -triage" mode increase the probability the unit will later enter " triage" mode conditions.

The fairness of limit setting decisions in our critical care unit may have been adversely affected by indirect communication. Until recently, very little has been known about communication in this milieu. Mielke et al found that ICU physicians communicated their bed allocation decisions and the reasons for them primarily to end user (referring) physicians. However, we found that communication of bed allocation decisions in our critical care unit was sometimes conducted through intermediaries, such as fellows, residents or receptionists. The effectiveness of communication conducted in this way, especially in regard to families is limited because family members often feel they have not been given enough time and require extra explanations if spoken to by junior physicians such as residents [12]. We observed that reasons for denial of an ICU bed were not directly communicated directly to families, who might learn of the decision from the referring service, as did those in Mielke's case study. Communication failures ("botch ups") were deemed the root cause of conflicts and appeals of decisions [4]. Bernstein et al also found that decisions and rationales were disseminated within the ICU and to end-users but not to patients, families or the public [10]. Families of critically ill patients may experience inadequate communication in close to half of their meetings with physicians, when these do occur. While there are many possible reasons, including the impact of anxiety and depression on family members' capacity to understand complex information about their loved one [13], physician related factors, such as meetings of short duration are also highlighted [14]. Even when surgical intensive care unit doctors do have meetings, they dominate the dialogue and utter more words per conversational turn than family members [15]. Within the critical care unit, the environment is noisy [16] and there are frequent disruptions by simultaneous events, which present problems not only for patients but also for researchers [17]. Our findings strengthen a chain of evidence showing how the publicity and appeals conditions of the leading fair process framework (accountability for reasonableness) may break down in critical care units [5]. They support recommendations that the publicity and appeals conditions can be improved by direct explanations of the reasons for admission to the intensive care unit and improved opportunities for debate about the appropriateness of bed allocation decisions.

Our case study methodology has some important limitations. Our choice of physician, nurse and administrative participants was guided by theoretical sampling. While this sampling method is systematic and non probabilistic, it identified initial participants comparable to those chose in previous investigations [4,10]. Although none of the participants we interviewed suggested that administrators should be included in the sample, their absence from our investigation limits our description because of the important mediation role some of them (such as the Medical Director on Call) played. We tried to ensure the validity of our analysis by giving interviewees transcripts of the analysis as it developed, but we did not do this systematically due to restrictions on health worker communication during the Toronto SARS epidemic [18]. Although this decreases the validity of our results, they are nonetheless congruent with other investigations in critical care units in ways we have already discussed. We continued to collect data until content "saturation" was encountered, but we did not interview family members of critically ill patients. This deliberate exclusion was felt to be justifiable because we were interested in how communication occurred between those who were officially responsible for decisions resulting in bed allocation in the critical care unit. However, the exclusion is unfortunate because it can be argued that family members represent an additional category of parallel track decision makers. For example, when disagreement over the appropriateness of life sustaining therapy occurs, our institutional policy mandates full treatment pending the outcome of a conflict resolution process. In effect this means that demands for critical care (even when judged medically inappropriate) from family members must be satisfied, even if for a short time by the allocation of a bed for the patient. Finally, our research setting in an urban, university affiliated teaching hospital may restrict the generalization of our results; communication in non-university teaching hospital contexts may involve participants who interact differently from those we observed.

## Conclusion

A formal policy guideline for communication of 'parallel track' decision making to the resource nurse should be created to ensure that independent decisions can be safely integrated within the resource constraints of the institution. Next, communication of such important decisions should not be delegated to third party intermediaries (e.g. Receptionists, Junior Housestaff) lacking meaningful involvement in the process. To improve the fairness of process in our institution, direct communication between the critical care physician and end-users would begin to satisfy the publicity condition of accountability for reasonableness. This seems especially important in relation to communication with the families of incapable patients affected by critical care unit bed allocation decisions.

## Competing interests

An earlier draft of this paper was awarded the 2003 K.J.R. Wightman Award for Research in Biomedical Ethics, Second Prize. The Royal College of Physicians & Surgeons of Canada has granted permission for publication of this material.

## Authors' contributions

ABC co-ordinated research group, created and maintained database, performed thematic analysis, composed manuscript and revisions

ASJ conducted participant interviews, transcribed tapes, performed thematic analysis

JG reviewed manuscript and gave suggestions for analysis and revisions

AHS reviewed manuscript and gave suggestions for analysis and revisions

DKM oversight and planning of research methodology, reviewed manuscript revisions

All authors read and approved the final manuscript.

## Pre-publication history

The pre-publication history for this paper can be accessed here:


